# Low-Dose Propofol with Peribulbar Anaesthesia for Cataract Surgery

**DOI:** 10.3390/jcm12072742

**Published:** 2023-04-06

**Authors:** Mahmoud Ahmed, Yamini Krishna, Petya Popova, Rose Herbert, Gediminas Sidaras, Anshoo Choudhary, Stephen B. Kaye

**Affiliations:** St Paul’s Eye Unit, Department of Ophthalmology, The Royal Liverpool University Hospital, Liverpool L7 8XP, UK

**Keywords:** PROMS, anaesthesia, sedation, cataract surgery, propofol

## Abstract

In this paper, we investigate the effect of sedation using low-dose propofol on patient reported outcome measures (PROMS) in patients undergoing cataract surgery. This is a randomised, single-blinded observational prospective study. Patients undergoing elective cataract surgery using peribulbar anaesthesia over consecutive cataract lists were selected for this trial. Patients were randomised to receive either no sedation or low-dose propofol (20 to 30 mg followed by 10 mg increments until the patient developed slurred speech alone) prior to the administration of local anaesthesia. Pain, satisfaction, anxiety, needle recall, pulse, and blood pressure (BP) were measured. A total of 97 patients were included, 50 of whom received propofol. There were 4 senior surgeons and anaesthetists. There were no ocular or systemic complications and all patients had uncomplicated surgery. Anxiety (*p* = 0.026), needle recall (*p* < 0.001), difference in systolic BP (*p* = 0.043), and pulse (*p* = 0.046) were dependent on patient age (*p* < 0.001) and the use of propofol (*p* = 0.007). Lower pain was associated with propofol (*p* = 0.008), as well as lower anxiety (*p* = 0.002), and increased patient age (*p* = 0.014). The administration of propofol was significantly associated with lower needle recall (*p* < 0.001), pre- to post-operative difference in systolic BP (*p* = 0.029), and mean BP (*p* = 0.044). Low-dose propofol given immediately prior to administration of local anaesthesia was associated with reduced pain and needle recall, as well as lower BP.

## 1. Introduction

Cataract surgery is the most common intraocular operation and is most often performed under local anaesthesia (LA) [1]. LA options include topical anaesthesia, retrobulbar, peribulbar, and sub-Tenon’s space blocks [2]. They are preferred to general anaesthesia in the elderly population due to medical co-morbidities. Surgery under LA, however, may be a stressful experience for the patient and administration of the LA itself can be associated with discomfort.

Sedation may be helpful with LA to lessen the experience of discomfort, movement and anxiety, which may in turn positively influence haemodynamic parameters, patient satisfaction, and overall improve surgical safety [2,3,4,5,6]. Use of sedation varies by location, being widely used in the USA, Canada, and Singapore [7] and rarely used (4.1% of cases) in the UK [8]. Oral and intravenous sedatives are used. The oral route demonstrated non-inferior results in patient satisfaction in a randomized control trial comparing oral triazolam with intravenous midazolam [9]. Sedatives used in cataract surgery include benzodiazepines, opioids, alpha-adrenoceptor agonists such as dexmedetomidine and ketamine, and propofol [10]. Nausea and vomiting, respiratory depression, post-operative confusion, and over-sedation are known side-effects. Dexmedetomine has shown promising characteristics of reduced intraocular pressure (IOP), improved patient satisfaction, and preferable analgesic and sedative effects in comparison with other agents, however, its administration as an intravenous infusion may limit its use in cataract surgery [7,11].

Patients are understandably anxious about undergoing cataract surgery under local anaesthesia and PROMS such as pain, satisfaction, needle recall, and anxiety are important measures of the success of cataract surgery. We found that increased levels of pain were associated with younger patients and increased pre-operative anxiety. The level of pain was lower with the use of propofol regardless of the surgeon or anaesthetist. Pain was associated with increased anxiety and reduced overall satisfaction. Anxiety can adversely affect haemodynamic parameters and since cataract surgery is typically performed in an ageing population with associated co-morbidities, many groups have tried using sedation to relax the patient, control patient haemodynamics, and minimise movement to favour the surgeon [1,3,4,5,6].

Previous studies have shown propofol to be an excellent choice for short-term sedation as a single low-dose bolus in comparison to other agents, due to its rapid induction, achieving adequate light sedation with anxiolytic effect whilst allowing the patient to remain co-operative. There is quick recovery to a clear-thinking state, with a low incidence of nausea and vomiting due to its rapid metabolism and mechanism of action, with few adverse effects [12]. Furthermore, propofol is less likely to cause patients to fall asleep during surgery and then suddenly move their head inadvertently upon waking [6,13,14].

Propofol is an ultra-fast-acting Gamma Aminobutyric Acid-A (GABA-A) receptor agonist which is administered as an intravenous bolus and is rapidly metabolised by the liver [12]. The dose can be titrated to produce sedative and anxiolytic effects [12]. It is most commonly used to induce general anaesthesia, however, it is also used in lower doses to maintain conscious sedation during procedures such as cataract surgery. Propofol has proven ideal for procedures requiring short sedation due to its rapid induction, and relatively quick recovery to a clear-thinking state with a low incidence of nausea and vomiting, possibly owing to the GABA-mediated inhibition of serotonin release at the chemoreceptor trigger zone [2,4,5,6,12,13,14,15]. Propofol can have haemodynamic and respiratory depressant effects depending on the dose administered, however, ease of dose adjustment helps to mitigate this. It is well known that propofol causes variable degrees of cardiovascular depression depending on patient related factors including patients’ age and co-morbidities. Propofol does not interact directly with antihypertensive drugs in general, but it can exaggerate their effect [16]. For patients on antihypertensive drugs, administering propofol should be done carefully, as the anaesthetist must assess the patient’s physiological status in relation to how much and how frequently propofol is administered.

A reduction in patient reported outcome measures (PROMS) such as pain, anxiety, and increased patient satisfaction are important measures of the quality of cataract surgery. We, therefore, investigated the effects of a single small bolus of propofol, ensuring the patient was still awake and co-operative, prior to administration of LA on haemodynamic parameters, and the following PROMS: anxiety, pain, satisfaction, and needle recall.

## 2. Materials and Methods

All consecutive adult patients undergoing elective cataract surgery over 5 theatre sessions on 5 separate days were included in this prospective study. Each theatre session had 2 theatre lists running concurrently. Patients had been randomly allocated to each of the theatre lists by waiting-list staff without involvement of the anaesthetists or surgeons. In one list, all patients received low-dose propofol prior to the anaesthetic block, whilst patients in the other list had no propofol. No pre-operative medication was prescribed. All patients had signed a written informed consent form and the study was conducted in accordance with the declaration of the procedure of Helsinki. Local Ethics Committee approval by the Institutional Board from the Royal Liverpool University Hospital was obtained with approval code 02062008 and trial registration number SP/RCT/08/anes/prop.

A single intravenous bolus of 20 to 30 mg propofol, dose determined by the patient’s build, was given by the anaesthetists followed by an increment of 10 mg every minute until the patient developed slurred speech only. The patient was otherwise awake and co-operative. A single transcutaneous peribulbar block of 5 mL dose (comprising 2% lidocaine, 5 mg/mL levobupivacaine, and 375 units hyaluronidase) was then administered to each patient. The same method of anaesthetic block was performed in all patients. Any ocular and systemic complications or adverse events or reactions from propofol and or the anaesthetic block were documented.

Blood pressure and pulse oximetry were measured pre- and post-operatively immediately following completion of the surgery by nurses who did not know which cohort the patient belonged to (single-blind). After surgery in the recovery area, each patient was given a questionnaire including a visual analogue scale, consisting of a 10 cm line, with two end points representing 0 (‘no pain’) and 10 (‘pain as bad as it could possibly be’), to record their perceived level of anxiety and pain prior to and during surgery followed by a satisfaction score and recall of the needle being inserted around the eye. Needle recall was recorded categorically as either ‘yes’ or ‘no’ and patient satisfaction with the anaesthetic as 1 of 4 options ranging from ‘very dissatisfied’ through to ‘very satisfied’.

Statistical analysis which was performed using SPSS (version 22). *p* < 0.05 was considered significant. A generalised linear model was used for discrete and categorical outcomes and a general linear model (SPSS 22) for the continuous and normally distributed dependent variable. Propofol, anaesthetist and surgeon were factors; age, pulse, systolic, diastolic, and mean blood pressure were covariates, with pain, satisfaction and anxiety scores, and needle recall were dependent outcome variables. Bland Altman plots were used to investigate the differences in pre- to post-operative blood pressure with the pre-operative measurement.

## 3. Results

A total of 97 patients (mean age 74.8 years) were included, 50 of whom received propofol. There were 4 anaesthetists and 4 surgeons. Adequate sedation was achieved in the propofol group, with the patients in a relaxed and co-operative state, responsive to verbal commands and tactile stimuli with all returning to a clear-thinking state by the end of the procedure. Time from the block being given to the end of the surgery ranged from 15 to a maximum of 20 min. There were no ocular nor systemic complications from propofol or local anaesthesia, and all patients had uncomplicated surgeries.

A summary of the results are included in Table 1 and Table 2.

### 3.1. Pain and Needle Recall

Increased pain was significantly associated with increased pre-operative anxiety (*p* = 0.002), a younger age (*p* = 0.014), and non-use of propofol (*p* = 0.010). Propofol use was associated with a lower needle recall (*p* < 0.001); 36% in the propofol group and 83% in the non-sedation cohort.

### 3.2. Anxiety

Post-operative anxiety was significantly associated with pain experienced (*p* < 0.001) during the procedure, pre-operative anxiety (*p* < 0.001), and age (*p* < 0.001). The corresponding change in the level of anxiety, pre- to post operatively, was significantly associated with the level of pre-operative anxiety (*p* < 0.001) and level of pain (*p* = 0.005) experienced.

### 3.3. Satisfaction

Patient satisfaction was high overall. The patients’ level of satisfaction, however, was significantly associated with the level of pain experienced during the procedure (*p* < 0.001), and the combination of surgeon and propofol use (*p* = 0.03), but not the anaesthetist (*p* = 0.24), patient’s age (*p* = 0.10), or pre-operative (*p* = 0.23) and post-operative anxiety scores (*p* = 0.40).

### 3.4. Blood Pressure and Pulse

**Figure 1:**
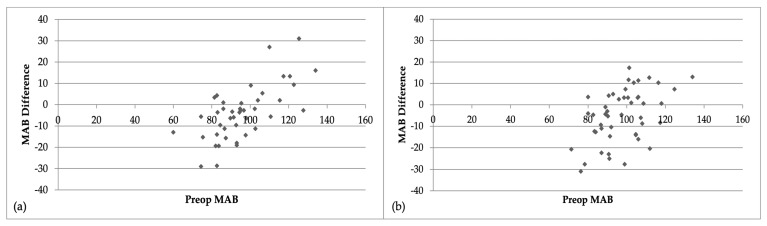
Bland Altman plots for mean arterial blood pressure (MAB) pre-operatively versus the difference [MAB pre-op minus MAB post-op] for (**a**) propofol group (R2 = 0.17, *p* = 0.003) and (**b**) the no propofol group (R2 = 0.23, *p* = 0.001).

Increasing age was significantly associated with a higher pre-op systolic BP (*p* = 0.024), reduced post-operative anxiety (*p* = 0.017) and pain (*p* = 0.016), as well as increased post-operative systolic (*p* = 0.02) but reduced post-operative diastolic BP (*p* = 0.02) and a reduced difference in pre-to post-operative pulse (*p* = 0.02). The changes in blood pressure are shown in Table 2. There was a significant reduction (11.5 mmHg; 17%) in the mean pre-operative (97.1 mmHg SD+ 15.5) to post-operative (85.6 mmHg SD +36.6) mean arterial blood pressure in patients receiving propofol, whereas there was an increase of 4.9 mmHg (23%) in those not receiving propofol (97.7 mmHg + 13.2 to 102.6 mmHg + 13.0). There were significant reductions in pre- to post-operative systolic and diastolic BP measurements within the propofol cohort (*p* < 0.01), which were also significantly greater than in patients who did not receive propofol (*p* < 0.01) (Figure 1). In comparison, there was an increase in both systolic (8.4 mmHg) and diastolic (3.1 mmHg) BP in those patients who did not receive propofol (Table 2).

## 4. Discussion

In this prospective, randomised, single-blinded study, we have shown that sedation using low-dose propofol can reduce the level of pain and effectively and safely reduce BP and pulse rate with all patients being co-operative and rapidly returning to a clear-thinking state. Interestingly, we used a lower dose compared to that used for sedation by previous investigators; yet the favourable effects continued beyond that expected for normal duration of action for propofol. Importantly, there were no adverse reactions despite the age group and concurrent co-morbidities.

Previous studies have also shown a reduction in BP following the administration of propofol, at higher doses than our study, and with variable lowering of heart rate, with only a few reported adverse but easily manageable cardiovascular disturbances [13,14,17,18,19]. Studies also demonstrated reduced needle recall [14,20]. Neel et al., observed a moderate reduction in intraocular pressure [19]. In addition to positively influencing and stabilising cardiovascular parameters, our study confirmed that low-dose propofol was well tolerated by patients, minimised patient discomfort and anxiety, and significantly reduced the recall of the peribulbar injection.

The current literature reports a good safety profile of low-dose propofol for conscious sedation in non-fasted patients undergoing cataract surgery [21,22,23]. Guerrier et al. found that a low dose of propofol is not associated with higher anaesthesia-related complications in a population of 8773 patients not fasting for cataract surgery [21]. Wiebe E. reports low-dose propofol has no reported respiratory risks such as desaturation or aspiration for the patient [22]. This is beneficial as most patients undergoing cataract surgery under local anaesthetic do not need to fast prior to their surgery. Pre-operative anxiety is worsened by hunger and thirst; thus, a non-fasting strategy is beneficial [23].

Sub-optimal anaesthesia such as eye movement or vitreous bulge, has been associated with an increase in ophthalmic surgical complication [2]. Avoidance of, for example, an increase in the patients’ blood pressure as has been achieved with the use of propofol, will therefore help optimise surgical conditions and reduce the risk of surgical complications. This would also be useful in patients who have anxiety-related high blood-pressured responses, thereby making surgery safer, reducing cancellation of surgery and further patient anxiety and stress.

There are limitations in this study. Although the 4 surgeons operated on patients with and without propofol, the use of propofol was limited to 2 of the 4 anaesthetists. The satisfaction scores were skewed to high, possibly as the patients were pleased to have had successful cataract surgery. The results pertain to peribulbar anaesthesia; however, it would be reasonable for the results to apply to sub-Tenon’s space local anaesthesia. Whether a similar benefit would apply to propofol use for topical anaesthesia is unclear.

This study helps demonstrate that low-dose propofol can be a helpful alternative when additional anaesthesia is deemed necessary by the operating surgeon and anaesthetic support is available. Although there are cost and resource implications for using sedation in patients undergoing cataract surgery, improving the patient experience and potentially reducing risk, are important considerations particularly in selected cases.

## Figures and Tables

**Table 1:** Comparative summary of results from both patient groups.

		Propofol	No Propofol
Age (years ± SD)		74.70 ± 10.03	74.20 ± 11.01
Age range (years)		45–91	37–91
Pre-operative pulse (bpm)	Mean ± SD	73.00 ± 12.21	78.00 ± 12.57
	IQR	17.25	20.00
Post-operative pulse (bpm)	Mean ± SD	66.50 ± 12.37	66.00 ± 10.42
	IQR	16.25	14.00
Pre-operative anxiety score	Mean	2.83	2.87
	IQR	5.00	5.00
Post-operative anxiety score	Mean	1.23	1.62
	IQR	1.00	2.00
Pain score	Mean	0.58	0.67
	IQR	0.75	1.00
Satisfaction score	Mean	3.94	3.89
	IQR	0.00	0.00
Peribulbar anaesthesia needle recall	Mean	0.38	0.83
	IQR	1.00	0.00

Bpm: beats per minute, IQR: interquartile range, SD: standard deviation.

**Table 2:** Comparative summary of the blood pressure results of both patient groups.

		Propofol	No Propofol
Systolic BP (mmHg)			
Pre-operative	Mean	139.4	142.4
	Median ± SD	134.00 ± 24.2	141.00 ± 23.7
	IQR	35.00	31.00
Post-operative	Mean	144.3	150.8
	Median ± SD	145.00 ± 22.3	144.00 ± 20.9
	IQR	32.00	36.00
Diastolic BP (mmHg)			
Pre-operative	Mean	75.4	75.4
	Median ± SD	75.50 ± 13.8	76.00 ± 11.5
	IQR	17.50	19.00
Post-operative	Mean	77.1	78.5
	Median ± SD	76.00 ± 11.1	77.00 ± 11.5
	IQR	14.00	18.00
Mean arterial blood pressure (mmHg)			
Pre-operative	Mean	97.1	97.7
	Median ± SD	94.84 ± 15.5	98.67 ± 13.2
	IQR	21.08	17.00
Post-operative	Mean	85.6	102.6
	Median ± SD	99.33 ± 12.0	101.3 ± 13.0
	IQR	13.3	20.3

BP: blood pressure, mmHg: millimeters of mercury, IQR: interquartile range, SD: standard deviation.

## Data Availability

Not applicable.
